# Improved Graph Convolutional Neural Network for Dance Tracking and Pose Estimation

**DOI:** 10.1155/2022/7133491

**Published:** 2022-06-27

**Authors:** Liangliang Zhang

**Affiliations:** ^1^Department of Music and Dance, Changzhi University, Changzhi, Shanxi 046011, China; ^2^School of Dance, Dankook University, Yongin, Gyeonggi-do 16890, Republic of Korea

## Abstract

Movement recognition technology is widely used in various practical application scenarios, but there are few researches on dance movement recognition at present. Aiming at the problem of low accuracy of dance movement recognition due to complex pose changes in dance movements, this paper designed an improved graph convolutional neural network algorithm for dance tracking and pose estimation. In this method, the spatial and temporal characteristics of motion are extracted from the skeleton joint diagram of human body. Then, GCN (graph convolutional neural) is used to extract potential spatial information between skeleton nodes. Finally, LSTM (long short-term memory) extracts the time series features before and after human actions as a supplement and performs late fusion of the prediction outputs of the two networks, respectively, to improve the problem of insufficient generalization ability of single network. The experimental results show that this method can effectively improve the accuracy of dance movement recognition in general movement recognition data set and dance pose data set. It has certain application value in dance self-help teaching, professional dancer movement correction, and other application scenarios.

## 1. Introduction

In the field of computer vision, human motion recognition is a hot research topic in recent years [1]. With the continuous exploration and research of scholars in the past decade, many excellent achievements have been made in human motion recognition, and this technology has been applied to all walks of life [2]. Each dance usually has some symbolic movements, gestures, props, etc., which can be used as key elements to record and classify each dance and can be used to build a database of a few dance movements and provide data support for more innovative applications [3].

In the field of dance research, some technical schemes for digital preservation and display of dance have emerged, but dance movements are almost recorded by large 3D motion capture equipment [4]. These devices are expensive and have poor flexibility, which affects the execution and recognition of actions. In addition, for the problem of occlusion in human movements, the motion characteristics in different scenes have still not achieved satisfactory effects, which also limits the inheritance and protection of dance [5]. For motion data collection, Kinect depth vision sensor equipment has the advantages of high resolution of depth map, low cost, and ability to directly track the motion trajectory of human skeleton [4]. Moreover, using skeleton information for motion recognition has two advantages. First, bone data is an abstraction of human body information in three-dimensional space, and it has certain robustness to noise such as background and light, thus providing a good representation of human behavior. Second, compared with RGB video data, bone data has a smaller data dimension, which also makes it possible to design lightweight and hardware friendly models.

With the deep integration of technology and culture, the application of computer vision technology in dance teaching has great potential [6]. General subjects follow the teaching mode of teachers speaking and students listening, but dance is performed by body, so teachers need to judge the standard degree of learners' movements and use different teaching methods and means. In traditional dance teaching, it is a major problem for learners to judge whether movements are standard or not [7]. Learners can only rely on their own subjective judgment and teacher's evaluation to judge whether their actions are standard or not. However, the traditional dance class has a large number of students and belongs to one-to-many teaching mode, so the teacher cannot give real-time guidance to every learner. In traditional dance teaching, although there is a unified standard of dance movements, the judgment of the standard of learners' dance movements mainly relies on subjective evaluation, lacking a quantifiable objective evaluation method. Applying information technology and exploring new teaching methods will provide infinite possibilities for the reform of dance teaching.

With the development of deep neural network, many human pose estimation methods based on deep neural network have been proposed in recent years. Top-down human posture estimation methods include CPM [8], Hourglass [9], Simple Baselines [10], and HRNet [11]. And bottom-up human pose estimation methods include OpenPose [12], DeepCut [13], and HigherHRNet [14]. However, there are few studies on motion similarity estimation, and most of them focus on motion recognition. For example, Zemike moment is used to describe the shape information of images in [15], and Support Vector Machine (SVM) is used to identify the movements of people. Literature [16] uses PAFs method to identify key information of human body and uses LSTM network to classify the information, so as to achieve the purpose of identifying human actions. In [17], the image is preprocessed and then modelled and analyzed to identify the actions of the characters. These methods only recognize the actions of the characters but do not judge whether the actions are standard or not and cannot provide suggestions for the improvement of the gestures for learners. Based on the above problems, this paper proposes a dual-flow network method based on the combination of GCN and LSTM, 2S-LSGCN.

This paper has three main contributions as follows.

1. GCN network is mainly used to extract spatial feature information implied in human skeleton.
2. Bi-LSTM was used to extract the time feature information of the actions in the complete skeleton graph as a supplement, and a time feature subsampling layer was added before input into the LSTM network to extract rich and abstract time sequence features.
3. The predicted outputs of the dual-flow network are, respectively, late fused to obtain the final predicted output value of the dual-flow network.

This paper consists of five main parts: the first part is the introduction, the second part is state of the art, the third part is methodology, the fourth part is result analysis and discussion, and the fifth part is the conclusion.

## 2. State of the Art

**Figure 1:**
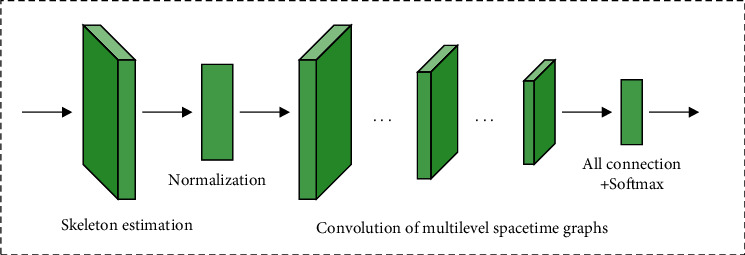
The architecture of the network.

**Figure 2:**
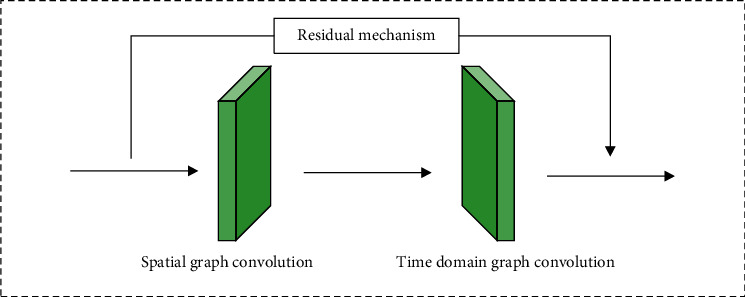
The architecture of a block.

A spatial-temporal Graph Convolutional neural Network (ST-GCN) based on bone data can extract spatial-temporal features from both time and space. The main process is as follows. Firstly, the graph structure data representing the bone sequence of a given action video is constructed and used as the input of ST-GCN. Then, a series of spatiotemporal convolution operations are performed to extract high-level spatiotemporal features. Finally, Softmax classifier is used to obtain the classification results. ST-GCN realizes end-to-end training, and its overall structure and convolution unit structure are shown in Figures 1 and 2.

Each graph convolution unit contains a spatial convolution layer, a temporal convolution layer, and a residual structure. There is a learnable edge weight parameter in the convolution unit of the space-time graph, which is used to learn the importance of edges between nodes. The advantage of this edge weight is that it can be used as an internal attention mechanism of ST-GCN to strengthen relevant information. However, the deficiency is that the key nodes and structural features are not effectively strengthened. ST-GCN time convolution layer adopts convolution operation with fixed structure, and the convolution kernel size is single definite value. Firstly, the spatial structure features are obtained through the spatial convolution layer, and then the time features in the direction of time flow are obtained through time convolution. Finally, the residual mechanism is used to fuse the original input and the spatiotemporal features of the high-level to form the output of each spatiotemporal convolution unit. In each ST-GCN space-time convolution unit, spatial graph convolution and temporal graph convolution can have similar expressions.

(1) $$\begin{array}{c} I_{\text{out}}\left(q_{nx}\right)=\sum_{q_{n}}\in H\left(q_{n}\right)\frac{1}{K_{nx}\left(q_{ny}\right)}I_{\text{in}}\left(q_{ny}\right)\cdot m\left(L_{nx}\left(q_{ny}\right)\right),\\ H\left(q_{nx}\right)=\left\{q_{vy}\left|d\left(q_{ny},q_{nx}\right)\leq Z,\right|v-n_{w}l\leq \left(\frac{R}{2}\right)\right\}. \end{array}$$

1/*K*_*nx*_(*q*_*ny*_) is the normalized term, *q*_*nx*_ represents the target node, and *q*_*ny*_ represents the neighbor node of *nx*. *M*() is the weight function. *H*(*q*_*nx*_) is the sampling function. *L*_*nx*_ represents the label graph with *q*_*nx*_ as the target center. *R* represents the size of time convolution kernel. *d*(*q*_*ny*_, *q*_*nx*_) is the shortest path from node *q*_*nx*_ to *q*_*ny*_. *Z* is the set policy parameter. *q*_*vy*_ represents the set of sampling nodes, *v* stands for sample frame, and *n*_*w*_ stands for target frame.

**Figure 3:**
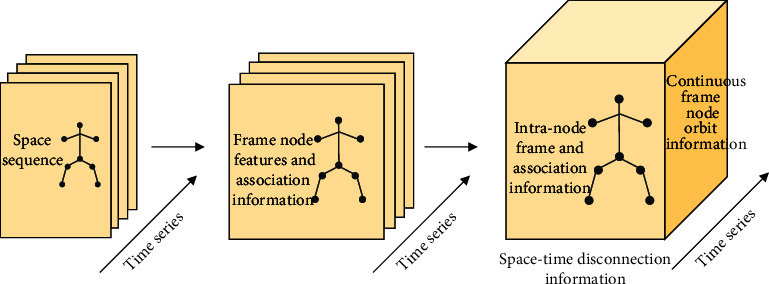
Spatiotemporal convolution flowchart.

The process of obtaining spatiotemporal correlation information through spatiotemporal graph convolution is shown in Figure 3. The input bone data contains two sequences: spatial sequence and time sequence. The spatial sequence is the sequence of key nodes in a single frame, and the time sequence is the sequence of time stream. Firstly, the structural features of each single frame are extracted from the spatial sequence through the spatial convolution layer. These features not only contain node features and the association information between nodes within the frame, but also retain the original time series. Then, interframe features are extracted from the direction of time stream through the time convolution layer. Interframe features include the track features of nodes, namely, time association information. Through the above two steps, spatiotemporal convolution obtains not only the structured spatial correlation information in a single frame, but also the temporal correlation information of node trajectories between consecutive frames. Thus, a feature map containing a large amount of spatiotemporal correlation information is formed.

## 3. Methodology

### 3.1. Acquisition of Human Skeleton Features

In the task of dance motion tracking and pose estimation of video stream, most researchers only focus on the pixel information in RGB video stream. It ignores the performance of human movements and is mainly completed by mutual traction and cooperation between skeleton and joints. Therefore, human skeleton joint diagram contains abundant information of action characteristics. However, most of the action recognition data sets, such as HMDB, 20BN-Jester, Kinetics, etc., have only RGB video or image samples and do not have the human point information.

At present, there are mainly two methods to obtain the characteristic information of human body joints in the sequence stream: ① To capture the depth information of the movement of people in the three-dimensional space through Kinect (3D motion camera) and then to obtain the coordinates of bone points from the depth image to form the joint diagram of human skeleton. ② The 2D pose estimation algorithm (such as Open) can be used for the RGB motion video stream (Pseo) to extract the 2D coordinates of the joints and bone information between the joints.

### 3.2. Graph Convolutional Neural Network

Since the human skeleton spatiotemporal graph input into the network is irregular non-European spatial data, the traditional convolutional network (CNN) cannot be directly applied to extract the features of the graph data, so graph convolution is used to extract the local location features of the node space. For the spatial dimension of skeleton space-time graph, the convolution operation of graph convolution operation for each vertex *q*_*x*_ is as follows:

(2) $$\begin{array}{c} f_{\text{out}}\left(q_{x}\right)=\sum_{qy}\in H_{x}\frac{1}{K_{xy}}f_{\text{in}}\left(q_{y}\right)\cdot M\left(l_{x}\left(q_{y}\right)\right), \end{array}$$

where *f*_out_ represents the feature structure, *q*_*y*_ represents the vertex in the graph, and *H*_*x*_ represents the receptive field of the convolution operation on *q*_*x*_ (the distance between the convolution center and its neighbor node is defined as 1). *M* is the weight function, similar to the traditional convolution (CNN).

### 3.3. LSTM Network

RNN has become a common method in video sequential tasks, and LSTM is the best RNN at present. Mainly due to its long time memory, when processing the sequence, the output features of this moment will be input together with the sample information of the next moment. In this way, the time information of the sequence is well preserved and the perceptual ability of the model to the information between the action frames is greatly improved.

LSTM is usually used to deal with time-dependent sequence problems, but LSTM's memory capacity is also limited, while Bi-LSTM has a stronger ability to deal with time series. Bi-LSTM uses two layers of LSTM connected in different directions to capture the deep spatiotemporal characteristics of three-dimensional bone coordinates evolving over time. The LSTM network consists of three gates (input gate *x*_*n*_, forget gate *f*_*n*_, and output gate *o*_*n*_), unit state *c*_*n*_, and hidden state *b*_*n*_. Among them, LSTM's ability to extract correlation information from time series mainly benefits from the clever design of unit state *c*_*n*_ and hidden state *b*_*n*_, which enables LSTM to choose to discard or retain features with temporal significance. The specific calculation is as follows:

(3) $$\begin{array}{c} f_{n}=\sigma_{a}\left(M_{f}i_{n}+P_{f}b_{n-1}+h_{f}\right),\\ x_{n}=\sigma_{a}\left(M_{x}i_{n}+P_{x}b_{n-1}+h_{x}\right),\\ o_{n}=\sigma_{a}\left(M_{o}i_{n}+P_{o}b_{n-1}+h_{o}\right),\\ c_{n}=f_{n}\circ c_{n-1}+x_{n}\circ \sigma_{c}\left(M_{c}i_{n}+P_{c}b_{n-1}+h_{c}\right),\\ b_{n}=o_{n}\circ \sigma_{b}\left(c_{n}\right). \end{array}$$

**Figure 4:**
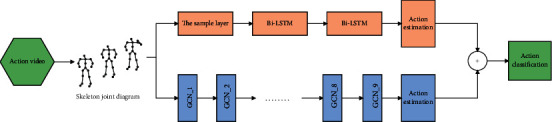
2s-LSGCN network structure.

2S-LSGCN is a combination of LSTM and GCN. The graph convolutional neural network (GCN) is used to extract the spatial relationship features of nodes in the skeleton graph, and the bidirectional long and short memory network (Bi-LSTM) with stronger time memory ability is used to extract the time feature information of action sequences in the skeleton graph. Specifically, the idea of the model is to use 2D attitude estimation algorithm to calculate the 2-dimensional coordinates (*i, j, c*) of the human body's key points in each frame space from the action video stream, where *i* and *j*, respectively, represent two-dimensional coordinates and *c* represents confidence degree. The skeleton joint graph composed of joint features in time and space was input into GCN network and LSTM network, respectively. As the time series of the original skeleton joints are too long and the duration of each video sample is different, LSTM network cannot effectively extract the feature relationship before and after the time series. The time dimension in the skeleton joint diagram was reduced by adding subsampled layers. Finally, the prediction output of dual-stream network is combined with late fusion to solve the problem of insufficient generalization ability of single network. The model in this paper is shown below (see Figure 4).

### 3.4. Skeleton Joint Diagram Construction

In this paper, the graph structure data-skeleton joint diagram is used as the input of LSTM and GCN, respectively. The following describes the construction method of skeleton joint diagram. Firstly, 2D pose estimation algorithm is used in this paper. Open Pseo is used to obtain the coordinate information of each frame node in the video, and the coordinates of different positions of each frame node are combined into the skeleton joint space-time map.

Specifically, all videos need to be adjusted to a smaller resolution (340 × 256) and converted to a 30FPS frame rate. The purpose is to improve the accuracy of attitude estimation and reduce the reasoning time of the model. Second, the Open Pseo toolbox was used to estimate the positions of the 18 joints in each frame. The toolbox provides 2D coordinates (*i, j*) in pixel coordinates and gives confidence scores *c* for 18 human joints. Therefore, we use the (*i, j, c*) tuple to represent the two-dimensional coordinate information of each joint.

**Figure 5:**
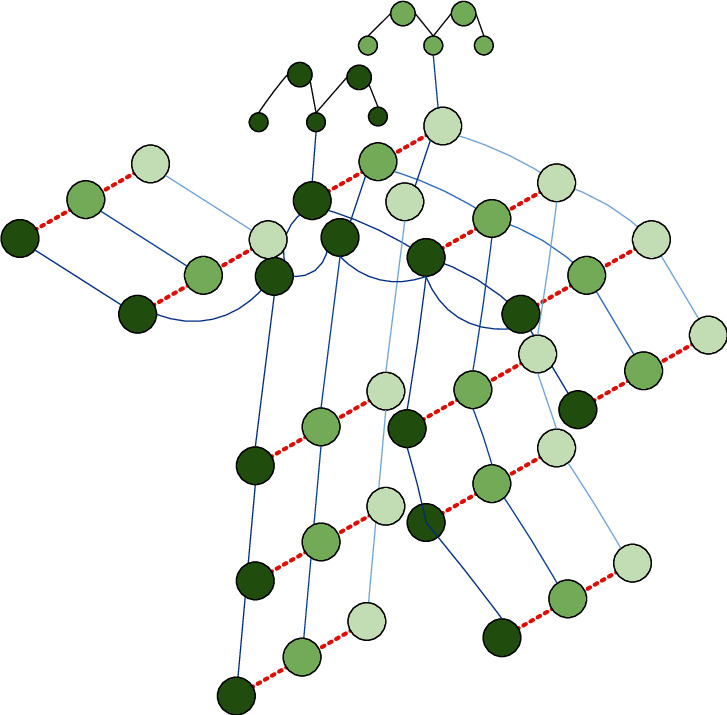
Spatiotemporal skeleton joints.

The 2D pose estimation algorithm only obtains the coordinate information of the joints, but the spatial and temporal dimensions between the joints are not connected and cannot be directly input into the graph convolutional neural network. Therefore, the complete skeleton joint map should be established according to the joint coordinates first.  Figure 5 shows the constructed spatiotemporal skeleton joint diagram, where the joints are represented as vertices and their natural connections in the human body are represented as edges.

### 3.5. Space GCN Network

Compared with action RGB video stream action, skeleton joint diagram has smaller feature dimension. Therefore, the network based on graph structure as input has not only doubled its running speed, but also far less computation than traditional CNN network. However, it also brings a difficult problem, that is, how to maintain high recognition accuracy under the condition of simple spatial topology and lack of time series information, that is, how to extract abstract high-dimensional features from a small number of low-dimensional features and then accurately predict the categories of actions. Starting from this problem, this paper uses multilayer stacked deep neural network, specifically stacking multiple graph convolution layers with different input and output dimensions and then extracting rich high-dimensional features. Secondly, the number of human nodes is generally less than 30, so the convolution kernel with a small scale of 3*∗*3 is used to greatly reduce the receptive field, and the joint information with strong spatial dependence can be extracted more centrally. Finally, the experimental results show that the prediction results of graph convolutional networks with less than 9 layers will be greatly reduced. When it is larger than 9 layers, the accuracy of the network does not improve, but the amount of computation increases exponentially. Therefore, the model determines the number of graph convolution layers at 9 layers.

There is no fixed number of neighbor nodes for each vertex in the skeleton space-time graph, so the convolution operation in the graph structure data needs to define the mapping function *l*_*x*_ so that each vertex corresponds to a unique weight vector. According to the results in ST-GCN, the optimal mapping function is obtained by defining the segmentation strategy according to the distance from the center of gravity. The specific calculation is as follows:

(4) $$\begin{array}{c} l_{x}\left(q_{y}\right)=\left\{\begin{array}{c} S_{1}\text{if}r_{y}=r_{x},\\ S_{2}\text{if}r_{y}<r_{x},\\ S_{3}\text{if}r_{y}>r_{x}. \end{array}\right. \end{array}$$

The strategy sets the size of the graph convolution kernel as 3, and the receptive field *H*_*x*_ is divided into three subsets: (1) the root node of *S*_1_ itself; (2) *S*_2_ centripetal subset, that is, the neighbor node is closer to the center of gravity; (3) the *S*_3_ centrifugal subset, that is, the neighbor node is farther from the center of gravity.

With the deepening of network depth, the gradient becomes smaller and smaller after multiple convolution and multiplication, and the problem of gradient dissipation appears. Moreover, the difference between the input dimension and the output dimension is too large, and the extracted feature is too abstract, which is far from the original feature information, leading to the lower accuracy of motion prediction. Therefore, skip Connect is introduced in this paper to fuse the input characteristics of the convolution layer with the output of this layer, and the calculation mode is shown in the formula below.

(5) $$\begin{array}{c} i_{l+1}=i_{l}+F\left(i_{1},M_{l}\right), \end{array}$$

where *i*_1_ and *i*_*l*+1_ represent the input and output features of graph convolution layer, respectively, *F* represents a series of nonlinear transformations in this layer, and *M*_*l*_ is the set of weight parameters. If the characteristic dimension of the output changes after the feature passes through the convolution layer, upsampling or downsampling should be added to the residual formula, depending on the specific situation. The modified formula is as follows:

(6) $$\begin{array}{c} i_{l+1}=b\left(i_{l}\right)+F\left(i_{l},M_{l}\right), \end{array}$$

in order to avoid overfitting in this layer and reduce the number of network parameters. The output features of graph convolutional network are globally pooled, and the final loss function is ReLu function. The input is nonlinear processing, and the prediction results are output.

### 3.6. Time LSTM Network

Short and long memory network is a recurrent neural network (RNN), which can remember the feature relationships of a long time series. Since graph convolutional neural network is only suitable for simple time convolution kernel, the time dimension of skeleton joint graph is processed. Therefore, only the features that change before and after some nodes are extracted, but the rich time information of video stream is lost. Therefore, bi-long and short memory network (Bi-LSTM) is used as a supplement for the model in this paper. Bi-LSTM can learn sequential and reverse time information at the same time, so as to enhance the model's ability to extract sequential information.

Different from the end-to-end CNN-LSTM network, the high-dimensional features extracted by CNN are input into LSTM. In the model presented in this paper, the Bi-LSTM network uses the original unprocessed skeleton joint space-time map as input, so it retains richer original temporal features. Specifically, BL-1 and BL-2 represent the first and second Bi-LSTM layers. Stack BL-1 and BL-2 layers together, and the output of BL-1 serves as the input of BL-2. It is expressed by the following formula:

(7) $$\begin{array}{c} f_{\text{out}}\left(i\right)=f_{HL-2}\left(f_{HL-1}\left(i,M_{1}\right),M_{2}\right), \end{array}$$

where *f*_out_ is the output of Bi-LSTM at the second layer, *f*_*HL*−1_(*∗*) is the feature extraction function of Bi-LSTM at the first layer, and *f*_*HL*−2_(*∗*) is the feature extraction function of Bi-LSTM at the second layer. *M*_1_ and *M*_2_ are the weight parameters of BL-1 and BL-2 layers, respectively.

Due to the length of time feature sequence in input original skeleton joint space-time diagram is too long, and the duration of each video sample is different. Take the running video V1 of 10 s as an example; extract an input sample representing the video {*i*_1_, *i*_2_, *i*_3_,…, *i*_*n*−1_, *i*_*n*_}, where *n* *=* 300, namely, the input time characteristic dimension *n* *=* 300. If the sample data is directly input into the Bi-LSTM network, the input dimension in the network must also be equal to the sample feature dimension. However, the memory capacity of the recurrent neural network is limited, and Bi-LSTM cannot learn the pre- and postcorrelation from such a long time feature as *n* *=* 300 and will greatly increase the weight number of the network and consume a lot of computing resources.

Based on the above problems, before the skeleton joint diagram is directly input into Bi-LSTM network, the subsampled layer is introduced in this paper. By downsampling the time dimension of samples, a time series with shorter time dimension and more abstract features is obtained. It is found that mean pooling and 1*∗*1 convolution are two common and effective downsampling strategies. Mean pooling has excellent effect on dimensionality reduction of image features. However, the biggest difference between graph structure data and Euclidean spatial data is that there are spatial topological relationships among vertices in the graph, and mean pooling will lose such important topological connections. Therefore, the 1*∗*1 convolution operation is adopted in this paper to conduct downsampling dimension reduction for the time dimension of skeleton joint graph.

### 3.7. Dual-Stream 2S-LSGCN Network

The GCN is fused with the improved Bi-LSTM network to form 2S-LSGCN dual-current network, which not only greatly improves the accuracy of recognition, but also is a powerful feature extraction network running in parallel and synchronism. GCN is used to extract spatial information between input skeleton nodes, and the improved Bi-LSTM is used to extract time-dependent temporal features, so as to supplement the deficiency of GCN in time sensitivity. The late fusion of the prediction results of the two networks is carried out to obtain the final forecast output value of the dual-stream network. Specifically, the final detection performance is improved by combining the detection results of GCN and Bi-LSTM networks through the Add Connection strategy. The specific formula is as follows:

(8) $$\begin{array}{c} j_{2S-LSGCN}=j_{ACT}+\alpha *j_{Bi-LSTM}. \end{array}$$

*j*_2*S*−*LSGCN*_ represents the final classification prediction result of dual-flow network. *j*_*GCN*_ and *j*_*Bi*−*LSTM*_ indicate the detection results of GCN and Bi-LSTM networks, respectively. *α* is a hyperparameter that can be adjusted by feedback of experimental results.

## 4. Result Analysis and Discussion

### 4.1. Experimental Steps and Settings

In this paper, the OpenPose method is firstly used to extract bone joint coordinates from the video, and the missing data and illegal values are processed through data cleaning. In order to enhance the generalization ability, the joint coordinates are normalized, simulated camera movement, data filling, and other operations to enhance the data. On this basis, the edge weight matrix of each video is calculated, and the edge weight matrix and bone data are input into the network for training.

### 4.2. Experimental Evaluation Based on Kinetics Data Sets

Using Kinetics data set 1, the proposed method was compared with five other motion recognition methods [18–22]. Kinetics' data training set consisted of 15,440 videos and test set consisted of 1,241 videos, each with a bone sequence length of 300 frames. 65 epochs are trained by default for each network. The random gradient descent of 0.1 learning rate is used for learning, and the learning rate of every 10 epochs is reduced by 0.01. The dimension E of the delay vector is set to 4 and the step *τ* is 1 when calculating the edge weight matrix. In this paper, top-1 and top-5 accuracy rates were used to evaluate the effectiveness of the experimental effect. The higher the accuracy rate, the better the recognition effect.

**Figure 6:**
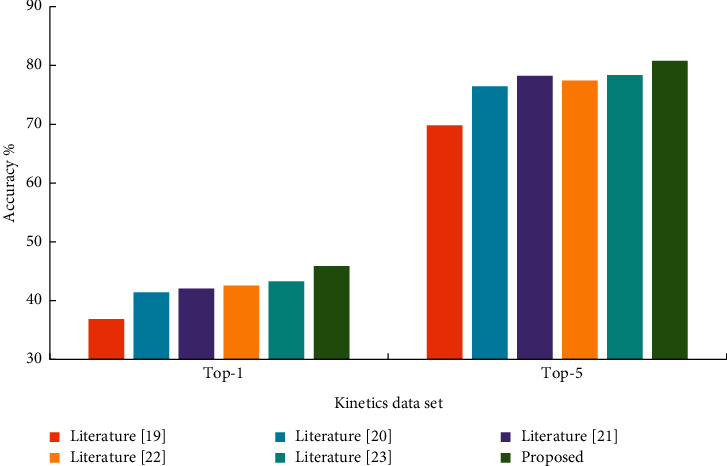
Experimental results of Kinetics data set.

As can be seen from Table 1 and Figure 6, due to the addition of edge weight causal correlation, the accuracy of actions in this paper is higher than other methods. Compared with the method in [18], top-1 and top-5 improved by 9% and 10.94%, respectively. Compared with [19], top-1 and top-5 improved by 4.44% and 4.32%, respectively. Compared with [20], top-1 was improved by 3.81% and top-5 by 2.54%. Compared with [21], top-1 and top-5 improved by 3.29% and 3.33%, respectively. Compared with [22], top-1 and top-5 improved by 2.6% and 2.41%, respectively.

As listed in Table 2, compared with the graph convolutional network model, the top-1 accuracy of the proposed method in some action classification is improved by 3% to 22%. Although recognition is slightly lower for certain actions such as kicking the hind and small jump combination. This is due to the uneven picture quality of Kinetics data sets, the low degree of specification of movements, and the fact that many movement categories are not strongly associated with physical movements. Since the graph convolutional network cannot capture the overall motion state, the recognition accuracy of actions that are closely related to human movement is higher, while that of actions that are closely related to human movement is lower.

### 4.3. Experimental Evaluation Based on Dance Data Sets

**Figure 7:**
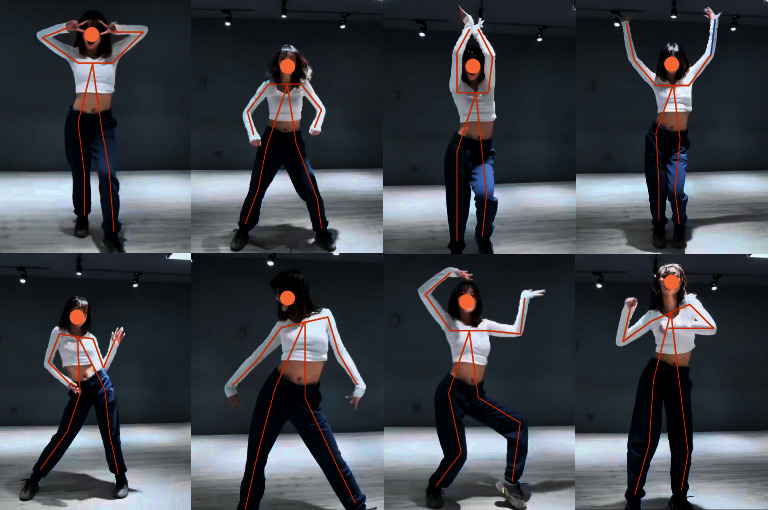
Example of modern dance data set.

This paper also constructs a real modern dance video data set. The data set, collected from 132 contemporary dance students, contains 2,956 video clips subdivided into 25 movements.  Figure 7 shows an example of a modern dance data set.

**Figure 8:**
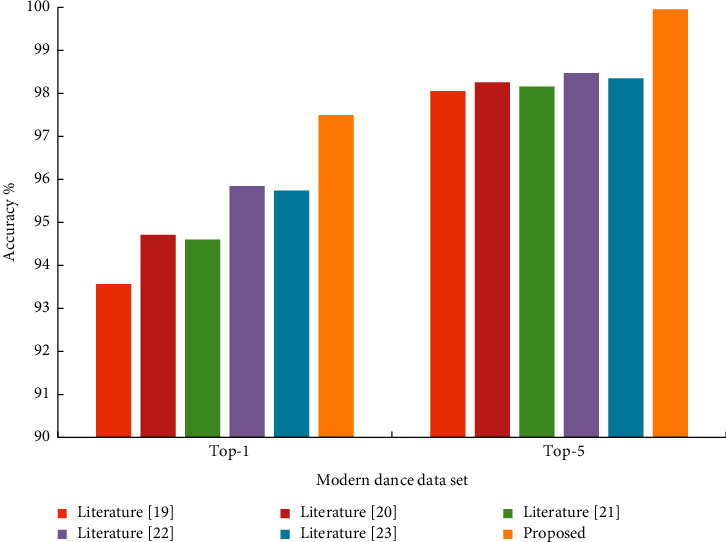
Comparison of recent models on modern dance data sets.

As shown in Table 3 and Figure 8, most models can achieve good recognition results in modern dance data sets.

The proposed method can achieve the performance for the following reasons:

1. The modern dance videos collected in this paper are all shot in front, and the subjects are always kept in the picture.
2. There are almost no extra objects blocking the human body and background interference in the video scene, which makes the extracted bone data have high integrity and accuracy. Integrity is very important for motion recognition.
3. Subjects perform modern dance routines with a high degree of completion and less nonstandard movements, which enables the network to better extract features. Due to the addition of causal coefficient as edge weight, this paper can highlight the main joints in the process of human movement, and its effect is still better than other methods. This shows that the graph convolution network based on joint causality is more biased towards some nodes. The model has been applied to the collection of modern dance movement library.

### 4.4. Convergence Performance Test

**Figure 9:**
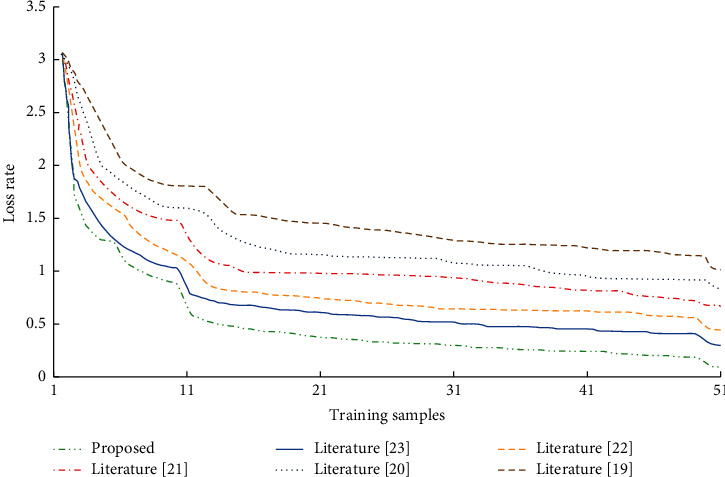
Convergence curve of loss function.

Figure 9 shows the comparison diagram of loss convergence between the other four models and the improved graph convolutional neural network model designed in this paper under the same parameter setting and training setting. It shows that, under the same training rounds, the convergence speed of multiscale spatiotemporal graph convolution model is faster than other models, and the effect is better.

## 5. Conclusion

Traditional dance teaching and training can only appraise whether its movements are standard through vision. It lacks objective quantified evaluation index problem. Combined with the practical application requirements of dance training, this paper proposes a dance tracking and pose estimation algorithm based on improved graph convolutional neural network, that is, a dual-flow network method based on the combination of GCN and LSTM. Unlike the traditional RGB image as input to the network, 2S-LSGCN uses as input a skeleton joint map composed of human joint coordinates. Then, GCN was used as a spatial feature extractor, and Bi-LSTM was used to extract temporal inverse information. A 1*∗*1 convolution subsampling layer is added to the sequential flow network to extract rich and abstract time features. Finally, the final forecast output value of dual traffic network is obtained by late fusion of the predicted output value of dual traffic network. Experimental evaluations on Kinetics public data sets and constructed real modern dance data sets demonstrate that the proposed method can effectively learn gesture features and improve the accuracy of dance movement recognition. In the following research, the problem of movement recognition for dancers with complex dance postures will be studied in more depth, and an intelligent dance-assisted training system with better performance is expected to be built.

## Figures and Tables

**Table 1:** Experimental results of Kinetics data set.

Kinetics data set	Top-1	Top-5
[18]	36.87	69.82
[19]	41.43	76.44
[20]	42.06	78.22
[21]	42.58	77.43
[22]	43.27	78.35
Proposed	45.87	80.76

**Table 2:** Top-1 accuracy of some kinetics actions.

Action category	ST-GCN	Proposed	Difference value
Pressing legs	37.7	44.3	6.6
Pressure shoulder	53	65.5	12.5
Push instep combination	19	17.5	−1.5
Stick training combination	76.5	81.1	4.6
Kick the hind legs	37	34	−3.0
Split jump	61	63.5	2.5
Big kick	17	15.8	−1.2
Lower waist	67.9	72.9	5
Splits	23.5	45.5	22
Small jump combination	75	71.5	−3.5

**Table 3:** Comparison of recent models on modern dance data sets.

Modern dance data set	Top-1	Top-5
[18]	94.67	99.16
[19]	95.82	99.37
[20]	95.71	99.27
[21]	96.95	99.58
[22]	96.85	99.46
Proposed	97.38	99.79

## Data Availability

The labeled data set used to support the findings of this study is available from the corresponding author upon request.

## References

[1] Zhang F., Wu T. Y., Pan J. S., Ding G., Li Z. (2019). Human motion recognition based on SVM in VR art media interaction environment. *Human-centric Computing and Information Sciences*.

[2] Ding C., Zhang L., Gu C. (2018). Non-contact human motion recognition based on UWB radar. *IEEE Journal on Emerging and Selected Topics in Circuits and Systems*.

[3] Tan B., Yang F. (2021). Dance movement design based on computer three-dimensional auxiliary system. *Computer-Aided Design and Applications*.

[4] Protopapadakis E., Voulodimos A., Doulamis A., Camarinopoulos S., Doulamis N., Miaoulis G. (2018). Dance pose identification from motion capture data: a comparison of classifiers. *Technologies*.

[5] Zhang Y., Zhang M. (2021). Machine learning model-based two-dimensional matrix computation model for human motion and dance recovery. *Complex & Intelligent Systems*.

[6] Iqbal J., Sidhu M. S. (2022). Acceptance of dance training system based on augmented reality and technology acceptance model (TAM). *Virtual Reality*.

[7] Georgios L. (2018). The transformation of traditional dance from its first to its second existence: the effectiveness of music-movement education and creative dance in the preservation of our cultural heritage. *Journal of Education and Training Studies*.

[8] Liang X., Gong K., Shen X. (2018). Look into person: joint body parsing & pose estimation network and a new benchmark. *IEEE Transactions on Pattern Analysis and Machine Intelligence*.

[9] Kim S. T., Lee H. J. (2020). Lightweight stacked Hourglass network for human pose estimation. *Applied Sciences*.

[10] Mehta D., Sridhar S., Sotnychenko O. (2017). VNect. *ACM Transactions on Graphics*.

[11] Wang X., Tong J., Wang R. (2021). Attention refined network for human pose estimation. *Neural Processing Letters*.

[12] Hernández Ó. G., Morell V., Ramon J. L., Jara C. A. (2021). Human pose detection for robotic-assisted and rehabilitation environments. *Applied Sciences*.

[13] Dang Q., Yin J., Wang B., Zheng W. (2019). Deep learning based 2D human pose estimation: a survey. *Tsinghua Science and Technology*.

[14] Ou Z., Luo Y. M., Chen J., Chen G. (2022). SRFNet: selective receptive field network for human pose estimation. *The Journal of Supercomputing*.

[15] Lou Y., Liu Y., Kaakinen J. K., Li X. (2017). Using support vector machines to identify literacy skills: evidence from eye movements. *Behavior Research Methods*.

[16] Qian K., Wu C., Yang Z., Liu Y., He F., Xing T. (2018). Enabling contactless detection of moving humans with dynamic speeds using CSI. *ACM Transactions on Embedded Computing Systems*.

[17] Nadeem A., Jalal A., Kim K. (2021). Automatic human posture estimation for sport activity recognition with robust body parts detection and entropy Markov model. *Multimedia Tools and Applications*.

[18] Oyedotun O. K., Khashman A. (2017). Deep learning in vision-based static hand gesture recognition. *Neural Computing & Applications*.

[19] Zhang L., Zhang S., Jiang F., Qi Y., Zhang J., Guo Y. (2017). BoMW: bag of manifold words for one-shot learning gesture recognition from kinect. *IEEE Transactions on Circuits and Systems for Video Technology*.

[20] Li Y., He Z., Ye X., He Z. (2019). Spatial temporal graph convolutional networks for skeleton-based dynamic hand gesture recognition. *EURASIP Journal on Image and Video Processing*.

[21] Zhang W., Lin Z., Cheng J., Ma C., Deng X., Wang H. (2020). Sta-gcn: two-stream graph convolutional network with spatial–temporal attention for hand gesture recognition. *The Visual Computer*.

[22] Wu Y., Liang S., Zhang L. (2018). Gesture recognition method based on a single-channel sEMG envelope signal. *EURASIP Journal on Wireless Communications and Networking*.

